# Heat Shock Proteins 27, 70, and 110: Expression and Prognostic Significance in Colorectal Cancer

**DOI:** 10.3390/cancers13174407

**Published:** 2021-08-31

**Authors:** Jan Hrudka, Karolína Jelínková, Hana Fišerová, Radoslav Matěj, Václav Mandys, Petr Waldauf

**Affiliations:** 1 Department of Pathology, 3rd Faculty of Medicine, Charles University, University Hospital Kralovske Vinohrady, 10000 Prague, Czech Republic; kar.jelinkova@gmail.com (K.J.); hanafiser.hf@gmail.com (H.F.); radoslav.matej@ftn.cz (R.M.); vaclav.mandys@lf3.cuni.cz (V.M.); 2 Department of Pathology and Molecular Medicine, 3rd Faculty of Medicine, Charles University, Thomayer University Hospital, 14000 Prague, Czech Republic; 3 Department of Pathology, 1st Faculty of Medicine, Charles University, General University Hospital, 12800 Prague, Czech Republic; 4 Department of Anesthesia and Intensive Care Medicine, 3rd Faculty of Medicine, Charles University, University Hospital Kralovske Vinohrady, 10000 Prague, Czech Republic; petr.waldauf@fnkv.cz

**Keywords:** colorectal carcinoma, heat shock protein, prognosis, survival, HSP27, HSP70, HSP110

## Abstract

**Simple Summary:**

Heat shock proteins (HSPs) are cytoprotective chaperones occurring in virtually all living organisms including various types of cancer cells to repair proteotoxic damage. Some of HSPs influence tumor prognosis and represent promising therapeutic targets. To clarify prognostic significance of HSPs 27, 70, and 110 in colorectal carcinoma, we retrospectively performed HSP immunohistochemistry on tissue microarrays from archive tumor tissue from 297 patients. Survival analysis revealed significantly shorter overall survival (OS) and borderline insignificantly shorter cancer specific survival (CSS) in patients with HSP70+ tumors. In HSP27+, both OS and CSS were insignificantly shorter. HSP110 showed no influence on survival. We found an association of HSP27 and HSP70 expression with advanced cancer stage. In multivariate analysis, only advanced stage and right sided localization were independent predictors of worse survival, whereas all three examined HSPs were insignificant. In conclusion, from all three HSPs examined in our study, only HSP70 had an impact on colorectal cancer prognosis, although it is stage-dependent.

**Abstract:**

Heat shock proteins (HSPs) are evolutionarily conserved chaperones occurring in virtually all living organisms playing a key role in the maintenance of cellular homeostasis. They are constitutively expressed to prevent and repair protein damage following various physiological and environmental stressors. HSPs are overexpressed in various types of cancers to provide cytoprotective function, and they have been described to influence prognosis and response to therapy. Moreover, they have been used as a tumor marker in blood serum biochemistry and they represent a potentially promising therapeutic target. To clarify prognostic significance of two canonical HSPs (27 and 70) and less known HSP110 (previously known as HSP105) in colorectal carcinoma (CRC), we retrospectively performed HSP immunohistochemistry on tissue microarrays from formalin-fixed paraffin-embedded tumor tissue from 297 patients with known follow-up. Survival analysis (univariate Kaplan–Meier analysis with the log-rank test and multivariate Cox regression) revealed significantly shorter overall survival (OS, mean 5.54 vs. 7.07, *p* = 0.033) and borderline insignificantly shorter cancer specific survival (CSS, mean 6.3 vs. 7.87 years, *p* = 0.066) in patients with HSP70+ tumors. In the case of HSP27+ tumors, there was an insignificantly shorter OS (mean 6.36 vs. 7.13 years, *p* = 0.2) and CSS (mean 7.17 vs. 7.95 years, *p* = 0.2). HSP110 showed no significant impact on survival. Using Pearson’s chi-squared test, there was a significant association of HSP27 and HSP70 expression with advanced cancer stage. HSP27+ tumors were more frequently mismatch-repair proficient and vice versa (*p* = 0.014), and they occurred more often in female patients and vice versa (*p* = 0.015). There was an enrichment of left sided tumors with HSP110+ compared to the right sided (*p* = 0.022). In multivariate Cox regression adjusted on the UICC stage, grade and right/left side; both HSPs 27 and 70 were not independent survival predictors (*p* = 0.616 & *p* = 0.586). In multivariate analysis, only advanced UICC stage (*p* = 0) and right sided localization (*p* = 0.04) were independent predictors of worse CSS. In conclusion, from all three HSPs examined in our study, only HSP70 expression worsened CRC prognosis, although stage-dependent. The contribution of this article may be seen as a large survival analysis of HSPs 27 and 70 and the largest analysis of HSP110 described in CRC.

## 1. Introduction

Colorectal carcinoma (CRC) is major issue regarding cancer related morbidity and mortality worldwide. In 2020, there were approximately 150,000 estimated cases in the United States, with a slowly decreasing mortality rate [1]. In 2018, CRC was the third most frequent malignancy and third most common cancer related cause of death among both men and women in the U.S. [2]. In the last two decades, there has been a strong need and significant development in terms of seeking new predictive markers and potential targets of biological therapy.

Heat shock proteins (HSPs) occur in virtually all living organisms from bacteria to humans, playing a pivotal role in the maintenance of cellular homeostasis. They are evolutionarily conserved molecules principally acting as chaperones. Their main function is to arrange appropriate intracellular protein folding and to prevent undesirable protein aggregation. In addition to being constitutively expressed (making up 5% to 10% of the total protein content under normal growth conditions), the synthesis of these proteins can be markedly induced (up to 15% of the total cellular protein content) by a range of cellular insults that cause protein unfolding, misfolding, or aggregation and a flux of newly synthesized non-native proteins, the function of which is to stabilize and refold proteins [3]. Expression of HSPs may be triggered by various environmental stressors: starvation, high temperature, freezing, radiation, water deprivation, and various chemicals; these basically promote the refolding of damaged proteins [3]. Moreover, HSPs interfere with immune T-cells [4] and they can inhibit apoptosis at both the mitochondrial and post-mitochondrial level [5,6]. HSPs are classified and numbered according to their molecular weight.

Cancer cells are subjected to various proteotoxic stressors including anticancer intervention-induced stress, immune response, elevated reactive oxygen species, enhanced hypoxic, and acidic conditions [7]. In response to physiological stress, cancer cells activate cytoprotective adaptive pathways in which HSP expression is upregulated. These have been described to influence various stages of malignant cell transformation and cancer progression in terms of cancer growth, invasion, metastasizing, and metabolism. Their overexpression has been described in various types of cancer including breast, ovarian, bladder, prostate, cervical, lung, esophageal, renal, and colorectal carcinoma [8] to play various prognostic roles. Moreover, they have been used as a tumor marker in blood serum biochemistry and represent a promising therapeutic target.

In the literature review, we found HSP27 and HSP70 to be described as the most important HSPs associated with CRC progression, whereas there are only sparse publications documenting the role of HSP110 in cancer but with references on promising results focused on CRC. In this study, we aimed to assess the prognostic significance of the three main HSPs described in relation to CRC in the literature (HSP 27, 70, 110), evaluating the correlation of HSP expression with the survival of patients suffering from CRC, and to discover the eventual link of HSP status to traditional clinico-pathological variables like grade, stage, anatomical site, variant morphology, and mismatch-repair (MMR) status.

## 2. Material and Methods

### 2.1. Patient Selection

A total of 297 patients with surgically treated histopathologically verified adenocarcinoma of the colon and rectum with known follow-up and formalin-fixed paraffin-embedded (FFPE) resection specimen tumor tissue available were found in the medical records of the pathology department. Conventional adenocarcinoma, mucinous adenocarcinoma, and signet-ring adenocarcinoma occurred among these cases. Grade and stage of the tumor was recorded based on medical records from the institution. Staging was classified according to TNM Classification from 2017 [9] with assignment of stage I–IV in accordance with the Union for International Cancer Control (UICC).

### 2.2. Microarrays

A tissue microarray (TMA) technique was used to make paraffin blocks for immunohistochemical slides using manual tissue arrayer TMA Master 3D Histech. From each paraffin block containing invasive adenocarcinoma tissue, two cylindrical samples measuring 2 mm were taken from random spots of the tumor tissue, without aiming to sample any specific area (center versus invasion front etc.). All samples were collected in a recipient paraffin TMA block. Each recipient block contained 20 samples from 10 cases.

### 2.3. Immunohistochemistry

For HSP immunohistochemistry, 4 µm-thick tissue sections were stained in a Ventana BenchMark ULTRA autostainer (Ventana Medical Systems, Tucson, Arizona). Monoclonal antibodies against HSP27 (BioSB, clone G3.1, 1:100), HSP70 (EmergoEurope, clone W27, 1:50), and HSP105/110 (AbCam, clone EPR4576, 1:200), MSH2 (clone G219-1129, Roche, ready to use), PMS2 (clone A16-4, Roche, ready to use), MSH6 (clone 44, Roche, ready to use), and MLH1 (clone M1, Roche, ready to use) were used. The positive reactions were visualized by the Ultraview Detection System (Ventana Medical Systems), counterstaining the slides with hematoxylin. Stained slides were dehydrated and covered in a xylene-based mounting medium.

### 2.4. Microscopy

**Figure 1:**
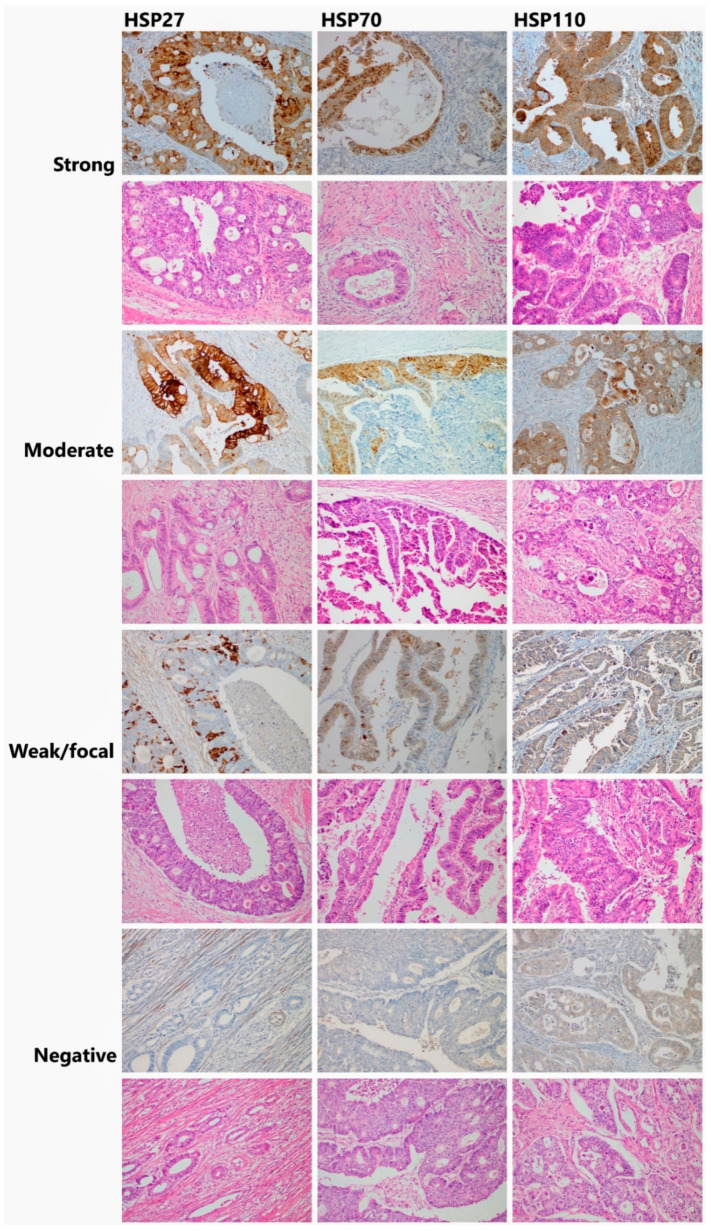
Histological images showing various conventional colorectal adenocarcinomas with different heat shock protein (HSP) staining intensity in immunohistochemistry.

A microscopical analysis of the immunohistochemistry slides was performed by two experienced routine surgical pathologists (JH and RM). In HSP27 and HSP70, cytoplasmic and nuclear staining were considered positive, respectively. The percentage of positive tumor cells was recorded (see Table S1). In HSP27 and HSP70, we used the cutoff value of ≥1% of stained cells to consider the tumor positive to obtain relevant numbers in both positive and negative cohorts. HSP110 showed more diffuse staining, which was categorized semi-quantitatively to negative/weak/moderate/strong (Figure 1). For statistics, we grouped negative and weak together as negative and moderate and strong as positive to binarize the cohort. Concerning MMR status, tumors with any apparent nuclear staining with MSH2, MSH6, PMS2, and MLH1 were considered MMR-proficient. Tumors with obvious loss of nuclear staining of anti-MMR antibodies with control positivity in stroma and lymphocytes were considered MMR-deficient.

### 2.5. Statistics

Overall survival (OS) was calculated from the date of surgery to date of recorded death or to the last known follow-up date (censoring). Cancer specific survival (CSS) was estimated from the date of the surgery to the date of cancer caused death; the patients with non-cancer related death were censored to the date of recorded death. For survival analysis, we performed a univariate Kaplan–Meier analysis with the log-rank test and confidence intervals calculated using the log-log method. Furthermore, we performed a multivariate Cox regression adjusting HSPs on binarized UICC stage, side, and grade (see below). The survival was analyzed independently from eventual administered radiotherapy and chemotherapy.

In the next step, survival analysis of all three HSPs was performed separately in right sided tumors (coecum, ascendens, hepatic flexure, transversum), in left sided tumors (lienal flexure, descendens, sigmoideum), and in the rectum. Furthermore, survival analysis was performed separately for MMR-proficient (MMR+) and MMR-deficient (MMR−) CRCs.

To assess eventual association of HSP expression with other examined parameters, Pearson’s chi-squared test was calculated to find eventual associations between binarized variables as follows: Localized tumor (UICC stage I + II) versus metastasizing (UICC stage III + IV), low grade (grade 1 + 2) versus high grade (grade 3), right sided tumors versus left sided tumors (including rectum), MMR+ versus MMR−, according to the morphology between adenocarcinoma not otherwise specified versus mucinous and signet ring carcinoma. Furthermore, we performed Kaplan–Meier survival analysis of the cohort according to these traditional factors.

*p* values < 0.05 were considered statistically significant. All analyses were performed in R version 4.0.3 (2020-10-10) [10]; survival analysis was conducted using package survival version 3.2–7 [11].

## 3. Results

All cases are listed in Table S1. Summary of the Pearson’s chi-squared test is presented in Table 1.

### 3.1. HSP27

**Figure 2:**
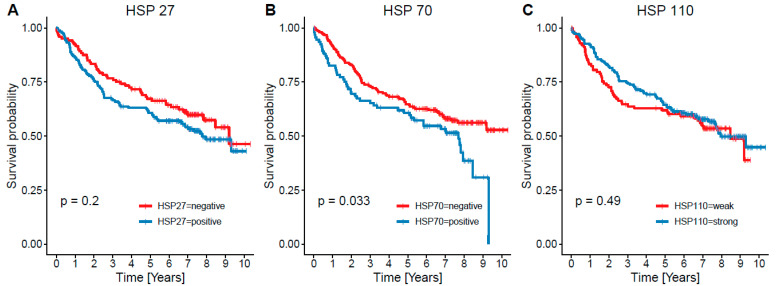
Kaplan–Meier curve documenting overall survival (OS) in relation to heat shock protein (HSP) status whereas only HSP27 shows a significant negative impact on survival. In HSP27, there was a visible trend, although insignificant.

**Figure 3:**
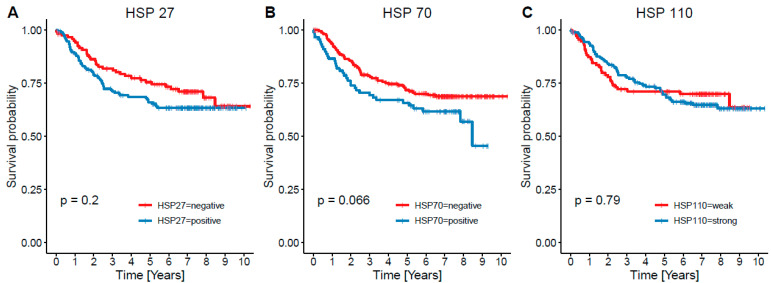
Kaplan–Meier curve documenting cancer specific survival (CSS) in relation to heat shock protein (HSP) status, both HSP27 and HSP70 displayed adverse effect on survival, but was insignificant.

Concerning OS independently from cause of death (Figure 2), there was an insignificant decrease of survival in patients with HSP27+ (*n* = 176) tumors compared to HSP27− (*n* = 121); (mean 6.36 vs. 7.13 years, *p* = 0.2). Evaluating OS in the right and left side and rectum separately, there were no significant results. CSS (Figure 3) was insignificantly shorter in HSP27+ (mean 7.17 vs. 7.95 years, *p* = 0.2); lower, but still insignificant *p* value was calculated in right sided (*n* = 119) HSP27+ (*n* = 65) tumors (mean 6.0 vs. 7.55 years, *p* = 0.94). Surprisingly, in rectal CRCs (*n* = 75) HSP27+ tumors (*n* = 57) displayed even better prognosis than HSP27− cases (mean 8.40 vs. 6.75 years, *p* = 0.2).

**Figure 4:**
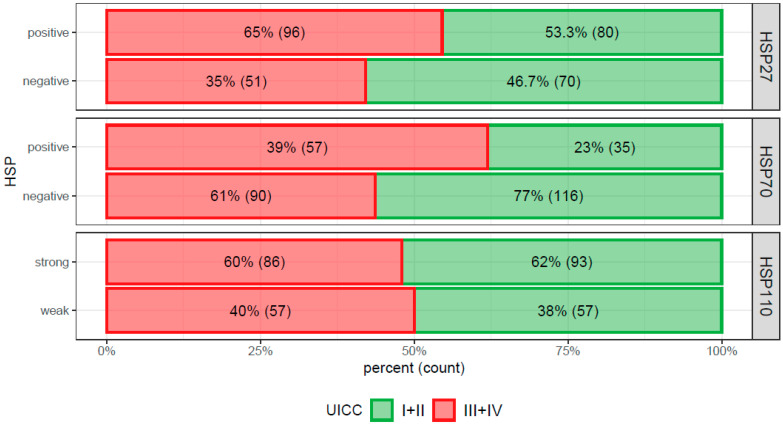
Bar chart documenting the Pearson’s chi squared test showing significantly more frequent expression of heat shock protein (HSP)27 (*p* = 0.048) and HSP70 (*p* = 0.005) in advanced stage colorectal cancer (Union for International Cancer Control III + IV stages). In the case of HSP110, there was no apparent association (*p* = 0.836).

**Figure 5:**
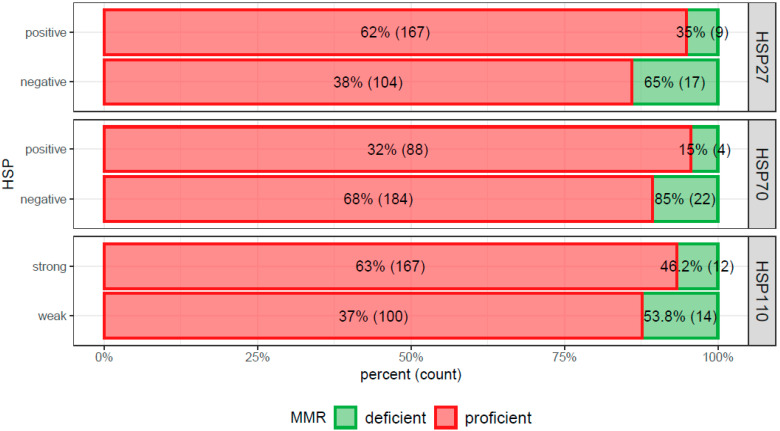
Bar chart documenting the Pearson’s chi squared test showing enrichment of mismatch-repair (MMR) proficient carcinomas with expression of heat shock proteins (HSPs)27 (*p* = 0.014), 70 (*p* = 0.117), and 110 (*p* = 0.153) whereas only HSP27 showed a significant result.

In the Pearson’s chi-squared test, HSP27+ carcinomas were relatively more frequent in female patients (*p* = 0.0150): there were 88 and 78 HSP27+/− tumors in male, but 88 and 42 HSP27+/− tumors in male, respectively. HSP27 expression showed a significant association with advanced UICC stages III + IV (*p* = 0.048): In stages I + II, 80 and 70 CRCs were HSP27+/−, and in stages III + IV, 96 and 51 CRCs were HSP27+/−, respectively (Figure 4). Moreover, HSP27+ tumors were prone to be more MMR+ (*p* = 0.014): among MMR+ 167/104 CRCs were HSP27+/− and among MMR− 9/17 CRCs were HSP27+/−, respectively (Figure 5). Pearson’s chi-squared test did not find any significant association with age, grade, side, and morphology.

Evaluating separately MMR+/MMR− cases, there was borderline insignificantly better CSS in HSP27 + MMR− (*n* = 9) and HSP27 − MMR− (17) cases compared to both HSP27 + MMR+ (*n* = 167) and HSP27 − MMR+ (*n* = 104) cases (mean 8.34 vs. 9.46 and 7.05 vs. 7.67 years, *p* = 0.063).

### 3.2. HSP70

In the case of HSP70, there was significantly shorter OS in patients with HSP70+ (*n* = 92) tumors in comparison with HSP70− (*n* = 206) CRCs (mean 5.54 vs. 7.07, *p* = 0.033) (Figure 2). Almost the same *p* value was reached in right sided tumors (HSP70+ *n* = 30, HSP70− *n* = 87, mean 4.52 vs. 6.5 years, *p* = 0.034), whereas survival analysis in left sided (HSP70+ *n* = 61, HSP70− *n* = 119) and rectal (HSP70+ *n* = 32, HSP70− *n* = 43) carcinomas showed no significant results. Evaluating CSS (Figure 3), there was borderline insignificant decrease in survival in HSP70+ cases (mean 6.3 vs. 7.87 years, *p* = 0.066). Like in OS, similar results were obtained in right sided HSP70+ tumors (mean 5.18 vs. 7.2 years, *p* = 0.074). Again, differences between left sided and rectal CRCs were insignificant.

In the Pearson’s chi-squared test, HSP70 positivity showed a significant association with advanced UICC stages III + IV (Figure 4): In stages I + II, 35 and 116 CRCs were HSP70+/−, and in stages III + IV, 57 and 90 CRCs were HSP70+/−, respectively (*p* = 0.004). In the case of HSP70+ tumors, they were slightly more frequently MMR+ and vice versa (Figure 5), but the association was not significant (*p* = 0.117). Pearson’s chi-squared test did not find any significant association with age, grade, side, and morphology.

If calculated MMR+/MMR− separately, there were significantly shorter CSS in HSP70 + MMR+ (*n* = 88) tumors compared to all the HSP70 − MMR+ (*n* = 184), HSP70 + MMR− (*n* = 4) and HSP70 − MMR− (*n* = 22) cases (mean 6.20 vs. 7.68 and 7.55 vs. 9.22 years, *p* = 0.028).

### 3.3. HSP110

Material from four cases was lost during tissue processing: 293 cases were evaluated with regard to HSP110. There was no significant difference concerning OS (Figure 2) and CSS (Figure 3) between no/weak (*n* = 114) and moderate/strong (*n* = 179) HSP110 expressing CRC; moderate/strong expressors displayed even longer OS (mean 6.88 vs. 6.09 years, *p* = 0.49) and CSS (mean 7.57 vs. 7.05 years, *p* = 0.79). In the same way, there were no significant differences when calculated for the right (*n* = 116) and the left (*n* = 178) side and rectum (*n* = 73) separately. Survival analysis of CSS for HSP110+/MMR− (*n* = 12), HSP110−/MMR- (*n* = 14), HSP110+/MMR+ (*n* = 167), and HSP110−/MMR+ (*n* = 100) evaluated separately also showed no significant result (mean 9.26 vs. 8.60 and 7.45 vs. 6.80, *p* = 0.097).

In the Pearson’s chi-squared test, there was a significant association of left sided carcinomas with HSP110 expression and vice versa (*p* = 0.022): there were 61 and 55 right sided HSP110+/− CRCs, but 118 and 59 HSP110+/− tumors, respectively. Pearson’s chi-squared test did not find any significant association of HSP110 expression with UICC stage (Figure 4), age, grade, morphology, or MMR-status (Figure 5).

### 3.4. Traditional Parameters

If sorted according to conventional prognostic factors, there was a significant negative impact of advanced UICC stage in terms of metastasizing tumors in stages III + IV (*n* = 147) vs. localized CRC in stages I + II (*n* = 150) on CSS (mean 6.03 vs. 8.89 years, *p* = 0). Patients with grade 3 (*n* = 67) CRCs showed significantly worse CSS compared to grade I + II (*n* = 212) tumors (mean 6.57 vs. 7.92 years, *p* = 0.043). Patients with right-sided (*n* = 117) tumors displayed shorter CSS in comparison to those with left-sided (*n* = 179) CRCs (mean 6.8 vs. 7.97 years, *p* = 0.045). Patients with MMR-deficient (*n* = 26) tumors had better CSS than those with MMR-proficient (*n* = 271) CRCs (mean 7.38 vs. 9.32, *p* = 0.012).

### 3.5. Multivariate Cox Regression

Adjusted on the UICC stage, grade and right/left side, both HSPs 27 and 70 were not independent survival predictors (*p* = 0.616 and *p* = 0.586). In multivariate analysis, only advanced UICC stage (*p* < 0.001) and right sided localization (*p* = 0.04) were independent predictors of worse CSS.

## 4. Discussion

To conclude the main significant findings of our study, there was a significant (*p* = 0.033) adverse prognostic role of HSP70 in colorectal cancer, predicting worse OS. To predict CSS, the HSP70 is clearly a negative marker, but borderline insignificant (*p* = 0.066) and stage-dependent. The patients with CRC expressing HSP27 showed both shorter OS and CSS, nevertheless, without statistical significance. Expression of HSP27 was associated with mismatch-repair proficient status and vice versa. Both HSP27 and HSP70 were associated with advanced tumor stage and were not significant predictors in multivariate analysis. Therefore, HSP27 and HSP70 may be regarded as hallmarks or epiphenomena of cancer growth and progression. HSP110 expression displayed no effect on survival.

HSP27 is a member of the small heat shock protein family gaining interest as a novel therapeutic option due to its role in apoptosis inhibition and enabling chemoresistance and cancer cell survival in stress conditions [12]. HSP27 has been referred to be overexpressed in cancers of the breast [13], stomach [14], endometrium [15], liver [16,17], and bladder [18] without unequivocal prognostic significance. It has been described to worsen prognosis of prostate adenocarcinoma [19,20,21], acute myeloid leukemia (AML) [22], meningioma [23], pulmonary adenocarcinoma, and colorectal carcinoma [4]; on the other hand, its positive prognostic effect was described in oral squamous cell carcinoma [24] and ovarian carcinoma [25].

The largest cohort studying a prognostic impact of HSP27 (and HSP60 and HSP70 as well, see later) was performed by Bauer et al. [26]. The authors found insignificantly decreased survival as observed in our cohort, with surprisingly similar *p*-value (*p* = 0.189 in Bauer’s study and 0.2 in ours). Bauer et al. described significantly shorter survival in patients with left-sided CRC, which was not the case in our study. In line with our results, there was a significant association of high HSP27 expression and advanced UICC stage. On the other hand, the authors described association of HSP27 expression and high tumor grade, which was not proven by us. Yu et al. described HSP27 as an independent predictor of worse prognosis on CRC when analyzing a sample of 182 patients, and they identified a significant correlation between HSP27 expression and TNM stage (*p* = 0.003). Patients with low HSP27 expression had better survival than those with high HSP27 expression. However, the authors found HSP27 positive immunohistochemistry in almost 80% of CRCs as they used a different method from ours, sorting HSP27 positivity according to staining intensity and not percentage of positive cells [27]. Tweedle et al. examined a relatively large cohort of 404 CRC patients, and surprisingly discovered the important negative prognostic value of HSP27 overexpression in rectal cancer (*p* = 0.006), but no effect of this in colonic cancer (*p* = 0.734): the authors explained this by the relative abundance of UICC stage III patients with rectal carcinoma in their cohort [28]. This differs markedly from our results, as we found slightly decreased survival in HSP27+ CRCs together whilst there was even better survival in rectum carcinomas expressing HSP27+ calculated separately. From our point of view, this discordance indicates HSP27 is not a very robust prognostic marker. Zhao described an association of HSP27 overexpression and lower occurrence of lymph node metastases in CRC, which is hard to explain—only 68 patients analyzed in this study may have provided misleading results [29].

Interestingly, HSP27 expression was more frequent in MMR proficient carcinomas and vice versa (*p* = 0.048). This may be explained by increased genomic and proteomic instability of MMR deficient tumors, therefore, cancer cells may fail to repair their proteins and resist environmental stress. This is in line with the generally better prognosis of MMR deficient CRCs, although HSP27 negativity is rather an epiphenomenon. To the best of our knowledge, this association has been described in HSP110 (see below, not corroborated by us), but not in HSP27 yet. The fact that HSP27 positive CRCs are prone to be rather MMR proficient is discordant with our finding of less frequent HSP27 negative CRCs in females, whereas MMR deficient CRCs are well known to occur mostly in women. This discordance is hard to explain.

HSP27 could be an important therapeutic target, especially in cancer, because it plays a significant role in cell apoptosis or multiple cellular pathways under stress conditions in cells [30]. HSP27 may be therapeutically downregulated by antisense oligonucleotides and small interfering RNA to HSP27, which may lead to chemotherapy enhancement and increased apoptosis [31], whereas HSP27 overexpression has been linked with increased chemoresistance in multiple cancer including breast, liver, melanoma, prostate, glioma, lung, gastric, pancreatic, kidney [30], and in CRC in vitro [32,33]. RP101 (Brivudine), Quercetin, Zerumbone, synthetic xanthone SW15, and peptide aptamers are examples of partly natural and partly synthetic molecules, which can interfere with HSP27 in sense of inhibition [34,35,36,37,38]. Anti-cancer effects of these molecules have been described in several cell lines [39,40,41,42,43,44,45,46,47,48]. Concerning clinical studies, RP101 (Brivudine) in combination with Gemcitabine positively influenced survival in patients with pancreatic cancer [37].

HSP70 is known to largely interfere with the caspase-dependent and independent pathways, causing inhibition of mitochondrial-mediated and death receptor mediated apoptosis [49]; moreover, it binds to several molecules (i.e., Akt), leading to cell survival signaling [4]. HSP70 has been found to be associated with the progression of several cancers: it stimulates migration and invasion of malignant cells in uterine cervix carcinoma [50] and bladder carcinoma [51]. Increased plasmatic levels of HSP70 worsen the prognosis of patients suffering from AML [52], and CRC [53]. Several immunohistochemical studies performed on FFPE tumor tissue documented negative prognostic value of HSP70 in the intestinal type of gastric cancer [54], and in non-small cell lung carcinoma [55]. Inversely, HSP70 expression has been described to be associated with better prognosis of oral squamous cell carcinoma [56], and esophageal carcinoma [57]. In line with this, Pfister et al. found better survival in patients with gastric cancer (*n* = 19), lung squamous cell carcinoma (*n* = 19), and CRC (*n* = 58) expressing HSP70 on the membrane in their study using flow cytometry, however, in relatively small cohorts [58]. The authors explained this phenomenon with increased tumor immunoreactivity. Inversely, patients with AML bearing membranous HSP70 expression had worse survival [59]. Concerning CRC, the HSP70 overexpression has been described in comparison to non-neoplastic large bowel mucosa [60]. With a focus on impact on survival, a recent meta-analysis of nine studies presented worse prognosis in CRCs expressing HSP70, in line with our results [61]. Bauer et al., as above-mentioned, found HSP70 to be an unfavorable prognostic predictor, in line with our findings. However, in their study, HSP70 was an independent marker with significantly low *p* value (*p* = 0.007) in multivariate analysis [26], whereas, in our study, this was a stage dependent result. Hwang et al. described increased HSP70 expression in cell lines from metastatic compared to non-metastatic CRC [62]. Interestingly, Cai et al. described prognostic significance of HSP70 in nasopharyngeal carcinoma depending on cellular compartmentalization of HSP expression: both membranous and cytoplasmic positivity improved prognosis in a relatively large cohort of patients (*n* = 507) whilst nuclear positivity showed negative impact on survival, with strikingly strong statistical significance [63]. However, this surprising finding is perfectly consistent with ours. The rationale for better prognosis of cancer with HSP70 on the membrane may be in the increased activity of the host immune system; on the other hand, cancer cells equipped with nuclear HSP70 expression may survive better under various proteotoxic stressors: this is very probably why patients with nuclear HSP70 expression in CRC have worse prognosis (shorter survival and advanced stage) in our study.

HSP110, previously called HSP105, is an example of high molecular HSP. It is distantly related to the HSP70 family and is involved in the prevention of aggregation during protein misfolding, providing thermal stability to cells, and in the stabilization of heat denatured proteins [4]. Buffering performance of HSP110 is significantly more efficient at recognizing denatured proteins than canonical HSP70 and thus, it is capable of salvaging misfolding-prone proteins during stress [7]. Its biological significance in cancer is less known in comparison to other HSPs. At the same time, it is known that HSP110 is abundant in CRC cells. Slaby et al. described an overexpression of HSP110 in CRC cells compared to non-neoplastic tissue in a molecular study of low-density oligonucleotide microarrays [64]. HSP110 enhances the CRC growth via STAT3 activation [65]. Moreover, HSP110 overexpression in CRC is thought to facilitate the pro-tumor anti-inflammatory setting of the macrophages [66]. Hwang et al. described increased HSP110 expression in cell lines from metastatic compared to non-metastatic CRC [62]. Kim et al. found better disease free survival in patients with microsatellite-instable (MMR deficient) CRC characterized by HSP110 downregulation; explaining this by deletion of the HSP110 T17 gene, which is associated with genomic instability in MMR deficient tumors [67,68]. The same was described in gastric cancer [69]. This was not corroborated by our results as there were only 26 MMR deficient CRCs as our study was not aimed solely at this particular subgroup, but there was no association of HSP110 with MMR status and no impact of HSP110 on prognosis. Nevertheless, there was significantly better survival of patients with MMR-deficient tumors, which is a well-known fact independently from HSP status [70,71].

The left-sided predominance of carcinomas expressing HSP110 found in the Pearson’s chi-squared test is difficult to explain. Only one third of HSP110 positive CRCs were in the right colon, whereas HSP110 positive tumors were generally more frequent than the HSP110 negative in our cohort. Nowadays, left and right sided CRCs are regarded as diseases with different genomic, molecular, and prognostic features. However, HSP110 enrichment of left sided CRCs has not yet been described in the literature and we do not possess a plausible explanation of this finding. We believe that HSP110 represents, rather, a drop in the ocean in the molecular complexity of colorectal carcinogenesis.

Apart from CRC, Kimura et al. performed a robust study on FFPE gastric cancer tissue identifying HSP110 expression as a marker of adverse prognosis [72]. On the other hand, HSP110 expression has been described to signify better prognosis in esophageal carcinoma [73]. Moreover, high expression of HSP110 has been found to be associated with worse prognosis of non-small cell lung cancer [74], and head and neck squamous carcinoma [75]; HSP110 overexpression has been described in hepatocellular carcinoma [76], and in malignant melanoma with relation to invasiveness [77]. In terms of therapy, to date, there are clinical trials with HSP105/110 peptide vaccine—the preliminary results suggest that the vaccine could induce immunological effects and improve prognosis in patients with CRC or esophageal cancer [78].

Like many other immunohistochemistry studies focused on HSPs, we had to consider several methodological issues. Various studies have used different systems of taking into account the tumor samples as positive or negative. To analyze sufficient numbers of patients in both the positive and negative group, we decided to binarize our cohort, similar to other studies focused on HSP expression in CRC [60,79]. Moreover, like all TMA analyses, the question of tumor heterogeneity cannot be ignored since this technique showed good correlation with whole section immunohistochemistry in a comparative study and, moreover, was previously validated in CRC [80].

In conclusion, from all three HSPs examined in our study (27, 70, 110), only HSP70 seems to be a relevant predictive marker of CRC prognosis, allowing some patient stratification, although it was not an independent predictor in the multivariate analysis adjusted on stage. Advanced stage (*p* = 0) and right-sided tumor site (*p* = 0.04) remained the only independent predictors of worse CSS. The worse prognosis of right-sided CRCs characterized by particular molecular properties is well-known and described [81]. The contribution of this article may be seen as a large survival analysis of three important HSPs described in CRC. As secondary findings, we report an association of HSP27 expression with MMR proficient status and male gender in CRC patients and enrichment of left-sided CRCs with HSP110 expression. Our research may serve as a document of the biological importance of HSPs 27 and 70 for cancer cell survival. Nowadays, a significant proportion of patients die due to metastatic CRC, therefore, more therapeutic trials of various HSP inhibitors in combination with chemotherapy are desirable. As shown by our article, some HSPs definitely help cancer cells to sustain and spread, therefore, patients may benefit from their inhibition. A study focused on the correlation of therapeutic response with tumor immunoprofile in terms of HSP would be of great interest. These particularly contradictory results show an urgent need for more studies in the field of the precise role and importance of HSPs in CRC for potential use as effective targets for new therapeutic strategies.

## Figures and Tables

**Table 1:** Summary of Pearson’s chi-squared test showing numbers of colorectal carcinomas (CRCs) expressing particular types of heat shock protein (HSP) in relation to traditional clinical-pathological variables. Significant *p* values (<0.05) are bold.

-	HSP27+	HSP27-	HSP70+	HSP70-	HSP110+	HSP110-
Age group	-	-	-	-	-	-
<50	12 (6.8%)	3 (2.5%)	6 (6.5%)	9 (4.3%)	11 (6.2%)	2 (1.8%)
50–59	28 (15.9%)	13 (10.8%)	17 (18.5%)	24 (11.7%)	29 (16.3%)	12 (10.5%)
60–69	56 (31.8%)	41 (34.2%)	29 (31.5%)	69 (33.7%)	57 (32.0%)	38 (33.3%)
70–79	49 (27.8%)	39 (32.5%)	21 (22.8%)	67 (32.7%)	48 (27.0%)	40 (35.1%)
80+	31 (17.6%)	24 (20%)	19 (20.7%)	36 (17.6%)	33 (18.5%)	22 (19.3%)
-	*p* = 0.307	-	*p* = 0.270	-	*p* = 0.179	-
Gender	-	-	-	-	-	-
F	88 (50%)	42 (35%)	41 (45%)	89 (43%)	80 (45%)	48 (42%)
M	88 (50%)	78 (65%)	51 (55%)	116 (57%)	98 (55%)	66 (58%)
-	***p* = 0.015**	-	*p* = 0.953	-	*p* = 0.722	-
UICC stage	-	-	-	-	-	-
I + II	80 (45.5%)	70 (58%)	35 (38%)	116 (56%)	93 (52%)	57 (50%)
III + IV	96 (54.5%)	51 (42%)	57 (62%)	90 (44%)	86 (48%)	57 (50%)
-	***p* = 0.048**	-	***p* = 0.005**	-	*p* = 0.836	-
Side	-	-	-	-	-	-
right	65 (37%)	52 (43%)	30 (33%)	87 (42%)	61 (34%)	55 (48.2%)
left	111 (63%)	68 (57%)	61 (67%)	119 (58%)	118 (66%)	59 (51.8%)
-	*p* = 0.325	-	*p* = 0.168	-	***p* = 0.022**	-
MMR	-	-	-	-	-	-
Deficient	9 (5%)	17 (14%)	4 (4%)	22 (11%)	12 (7%)	14 (12%)
Proficient	167 (95%)	104 (86%)	88 (96%)	184 (89%)	167 (93%)	100 (88%)
-	***p* = 0.013**	-	*p* = 0.117	-	*p* = 0.154	-
Grade	-	-	-	-	-	-
low grade (1 + 2)	124 (74%)	88 (79%)	68 (79%)	145 (75%)	131 (78%)	79 (74%)
high grade (3)	43 (26%)	24 (21%)	18 (21%)	49 (25%)	37 (22%)	28 (26%)
-	*p* = 0.493	-	*p* = 0.528	-	*p* = 0.520	-
Morphology	-	-	-	-	-	-
mucinous + signet ring	11 (6%)	7 (6%)	2 (2%)	16 (8%)	9 (5%)	7 (6%)
NOS	165 (94%)	114 (94%)	90 (98%)	190 (92%)	170 (95%)	107 (94%)
-	*p* = 1	-	*p* = 0.061	-	*p* = 0.885	-

## Data Availability

Research data are published online (Table S1).

## Supplementary Materials

The following are available online at https://www.mdpi.com/article/10.3390/cancers13174407/s1, Table S1: List of all analysed cases with examined variables.
